# Modeling the interplay between the HIF-1 and p53 pathways in hypoxia

**DOI:** 10.1038/srep13834

**Published:** 2015-09-08

**Authors:** Chun-Hong Zhou, Xiao-Peng Zhang, Feng Liu, Wei Wang

**Affiliations:** 1 National Laboratory of Solid State Microstructures and Department of Physics, Nanjing University, Nanjing 210093, China; 2 School of Physics and Electronic Engineering, Jiangsu Normal University, Xuzhou 221116, China; 3 Kuang Yaming Honors School, Nanjing University, Nanjing 210093, China; 4 Collaborative Innovation Center of Advanced Microstructures, Nanjing University, Nanjing 210093, China

## Abstract

Both the hypoxia-inducible factor-1 (HIF-1) and tumor suppressor p53 are involved in the cellular response to hypoxia. How the two transcription factors interact to determine cell fates is less well understood. Here, we developed a network model to characterize crosstalk between the HIF-1 and p53 pathways, taking into account that HIF-1α and p53 are targeted for proteasomal degradation by Mdm2 and compete for binding to limiting co-activator p300. We reported the network dynamics under various hypoxic conditions and revealed how the stabilization and transcriptional activities of p53 and HIF-1α are modulated to determine the cell fate. We showed that both the transrepression and transactivation activities of p53 promote apoptosis induction. This work provides new insight into the mechanism for the cellular response to hypoxia.

Hypoxia, a decrease in oxygen availability, affects both physiological development and tumorigenesis^1^. A key mediator of the cellular response to hypoxia is the hypoxia-inducible factor-1 (HIF-1), which is a heterodimer of *α* and *β* subunits. HIF-1β is constitutively expressed, while HIF-1α is regulated in an oxygen-dependent manner^2^. HIF-1α is inactive and remains at low levels in normoxia. With enough oxygen available, the hydroxylation of HIF-1α by PHD (prolyl hydroxylase domain protein) promotes its degradation by pVHL (von Hippel-Lindau protein)^3^, and the hydroxylation of HIF-1α by FIH-1 (factor inhibiting HIF-1) represses its transcriptional activity via preventing the recruitment of co-activator p300^4^. In hypoxia, both PHD and FIH-1 are deactivated, and thus HIF-1α is stabilized and activated. As a transcription factor, HIF-1 induces expression of target genes such as VEGF (vascular endothelial growth factor), EPO (erythropoiesis) and p21^5^,^6^. VEGF and EPO regulate adaptive responses to hypoxia, while p21 induces cell-cycle arrest.

The tumor suppressor p53 also mediates the hypoxic response. p53 is kept at basal levels in unstressed cells because of Mdm2-mediated ubiquitination and proteasomal degradation. The p53 response to hypoxia is diverse, depending on the cell type, the degree and duration of hypoxia^7^. For example, p53 is stabilized via phosphorylation by the ATR (ataxia-telangiectasia mutated and Rad3-related) kinase only under severe hypoxia^8^. Moreover, high levels of p53 can induce apoptosis in a different manner from that when it is activated by DNA damage^9^,^10^. Notably, different mechanisms have been proposed for p53-mediated apoptosis in hypoxia: p53 promotes apoptosis mainly by transrepressing antiapoptotic genes like microRNA(miR)-17-92^11^,^12^, or by transactivating proapoptotic genes such as *puma* (p53-upregulated mediator of apoptosis)*, fas* and *bnip3l*^13^,^14^,^15^. An issue naturally arises concerning whether these mechanisms are mutually exclusive or can be coordinated in one setting.

There exists an intricate interplay between p53 and HIF-1α. It was proposed that p53 stabilization is dependent on HIF-1α^16^; HIF-1α may bind to Mdm2, inhibiting Mdm2-dependent degradation of p53^17^. But HIF-1α accumulates with similar kinetics under mild and severe hypoxia, whereas p53 stabilization occurs only in severe hypoxia^8^. That is, HIF-1α upregulation is insufficient for p53 stabilization. On the other hand, it was reported that moderate p53 expression results in attenuated transcriptional activity of HIF-1 since p53 and HIF-1α compete for limiting p300, whereas high p53 expression leads to degradation of HIF-1α^18^. It is necessary to further clarify how the stabilization and transcriptional activities of p53 and HIF-1α are regulated under various hypoxic conditions.

Several mathematic models were developed to characterize the dynamics of the HIF-1 pathway, focusing on the mechanism for HIF-1α activation^19^,^20^,^21^. Few modeling studies explored the role for p53 in hypoxia. It is a challenge to reveal how the interplay between HIF-1α and p53 determines the cellular outcome. Here, we built a network model to characterize crosstalk between HIF-1 and p53 signaling, comprising their upstream modulators and downstream effectors. The analysis of network dynamics reveals that the cell either adapts to hypoxia or commits apoptosis, depending on the intensity of hypoxia. In mild hypoxia, HIF-1α accumulates to evoke transient cell-cycle arrest by inducing p21, whereas p53 remains at low levels. Under severe hypoxia, the accumulation of p53 attenuates HIF-1α activity and suffices to repress the expression of miR-17-92; consequently, BIM is induced to activate Caspase-3 and apoptosis ensues. In anoxia, p53 rises to high levels, transrepressing miR-17-92 and transactivating PUMA synergistically, whereas HIF-1α is degraded and inactivated. Thus, apoptosis can be triggered promptly. This work sheds new light on the mechanisms for the p53-HIF-1α interplay and hypoxia-induced apoptosis.

**Modeling crosstalk between the HIF-1 and p53 pathways.** We constructed an integrated model to explore the cell-fate decision upon hypoxia, characterizing the activation of HIF-1α and p53, their selective expression of target genes, and apoptosis induction (Fig. 1). We focused on the interplay between HIF-1α and p53 under various hypoxic conditions. The key points of the model are presented as follows.

**Regulation of HIF-1α activity.** In normoxia, HIF-1α is kept at low levels due to oxygen-dependent hydroxylation that promotes its degradation by pVHL^3^. HIF-1α can also be degraded by Mdm2 in a p53-dependent manner^22^. Under hypoxic conditions, the hydroxylation of HIF-1α drops remarkably, and HIF-1α accumulates. HIF-1α dimerises with HIF-1β in the nucleus. p300/CBP promotes the acetylation of HIF-1α, enhancing its transcriptional activity. In our model, HIF-1α is divided into unacetylated (HIF-1α, inactive) and acetylated (HIF-1α_ac_, active) forms, and their conversion is controlled by p300. The amount of p300 is limited^18^ and is set to a constant in the standard parameter setting. Because they can bind to p300 at different sites^23^,^24^, HIF-1α and p53 compete for limiting p300, the modeling of which is described later. Given the inhibitory effect of acetylation on protein degradation, only unacetylated HIF-1α is degraded through the PHD- or Mdm2-dependent mechanism^22^. The degradation and (de)acetylation processes are taken as enzyme-catalyzed reactions and assumed to follow the Michaelis-Menten kinetics (see Eqs. 1–2 in Supporting Material).

Activated HIF-1 can induce production of VEGF, EPO, PHD and p21. We explicitly characterize the induction of p21 and PHD with the Hill function (Eqs. 3 and 4), while implicating the roles for VEGF and EPO in the cellular adaptation to hypoxia. The HIF-1-PHD negative feedback promotes the adaptation of cells to mild hypoxia^25^,^26^. PHD is divided into inactive (PHD) and active (PHD_a_) forms, and their conversion depends on oxygen concentration (Eq. 6).

**Activation of ATR.** In severe hypoxia, the ribonucleotide reductase activity declines, leading to production of single-stranded DNA (ss-DNA) at stalled replication forks. ss-DNA is then coated with replication protein A (RPA), and the ATR-ATRIP (ATR interacting protein) complex is recruited to ss-DNA, which promotes ATR phosphorylation at T1989^27^. ATR phosphorylation enhances its interaction with TopBP1, which further activates the kinase activity of ATR. Thus, the activation of ATR is positively regulated.

ATR is divided into ATR (inactive) and ATR_p_ (active), and their dynamics are described by Eqs. 7–8. The total level of ATR is assumed to be constant since ATR is mainly regulated posttranslationally^27^. The phosphorylation and dephosphorylation of ATR are characterized by the Michaelis-Menten kinetics^28^. The parameters are set to ensure that most of ATR is inactive in normoxia or mild hypoxia and ATR is quickly activated under severe hypoxia^29^.

**Regulation of p53 activity.** p53 is degraded by Mdm2, which is induced by p53. ATR_p_ inhibits Mdm2 activity by phosphorylating it at Ser407^30^ and disrupts the p53-Mdm2 interaction by phosphorylating p53 at Ser15, thereby leading to p53 accumulation^29^. Based on its posttranslational modifications, nuclear p53 is divided into three forms: p53 (unphosphorylated), p53_p_ (phosphorylated), and p53_pac_ (phosphorylated and acetylated). Four forms of Mdm2 are defined here: Mdm2_c_ (cytoplasmic unphosphorylated), Mdm2_cp_ (cytoplasmic phosphorylated), Mdm2_n_ (nuclear unphosphorylated), and Mdm2_np_ (nuclear phosphorylated). For simplicity, we assume that only Mdm2_cp_ can enter the nucleus^31^. Mdm2_np_ cannot act as an E3 ubiquitin ligase^30^.

The coactivator p300 is required for the full transcriptional activity of p53 and HIF-1. Their competition for binding to p300 is characterized by the Michaelis-Menten kinetics with competitive inhibition (see Eqs. 1–2 and 10–11). The acetylation rate of p53_p_ (or HIF-1α) is an increasing function of its own concentration, and is a decreasing function of the level of HIF-1α (or p53_p_). On the other hand, p53, p53_p_ and HIF-1α are targeted for degradation by Mdm2. The involved competition is also characterized by the Michaelis-Menten kinetics (Eqs. 1, 9 and 10). Notably, Mdm2-mediated degradation of p53_pac_ is neglected since its dual modifications block their interaction. p53-induced expression of target genes, including *mdm2* and *puma*, is all characterized by the Hill function, and the Hill coefficient is set to 4 given the p53 tetramer acts as a transcription factor.

Mdm2_c_ can be phosphorylated by Akt, promoting its nuclear entry^32^. Akt is activated via phosphorylation by PIP3 (phosphatidylinositol 3,4,5-trisphosphate), which is dephosphorylated by PTEN (phosphatase and tensin homolog) into PIP2 (phosphatidylinositol-4, 5-bisphosphate). The phosphorylation and dephosphorylation of Mdm2, Akt and PIP2/3 are all characterized by the Michaelis-Menten kinetics (Eqs. 12–15, 17 and 19). The total amount of Akt and that of PIP2 and PIP3 are separately assumed to be constant, similar to ref. 33.

**Induction of apoptosis by p53.** Different mechanisms have been proposed for p53-induced apoptosis in response to hypoxia. On one hand, repression of miR-17-92 by p53 is important for apoptosis induction since this relives some proapoptotic genes such as *pten* and *bim* from the inhibition by miR-17-92^12^,^34^. FOXO (forkhead box protein class O) is a typical transcription factor of *bim*, and its transcriptional activity is inhibited indirectly by Akt via phosphorylation^35^. On the other hand, p53 also induces apoptosis by directly transactivating some proapoptotic genes such as *puma* and *fas*^13^,^14^.

Here, we assume that p53 indirectly upregulates BIM and PTEN by transrepressing miR-17-92 in severe hypoxia or anoxia and directly induces PUMA in anoxia. There are two forms of FOXO: FOXO (active) and FOXO_p_ (phosphorylated, inactive), and Akt promotes the conversion from FOXO to FOXO_p_. High expression of either BIM or PUMA promotes the release of cytochrome c (CytoC) from mitochondria, resulting in the activation of Caspase-3 (Casp3)^36^. A positive feedback exists between CytoC release and Casp3 activation^37^. The dynamics of CytoC and Casp3 are characterized in a similar manner to that in ref. 9 (Eqs. 26 and 27). The sustained activation of Casp3 is taken as the marker of apoptosis here.

## Results

### Overview of the dynamics of the HIF-1α and p53 pathways

**Figure 2:**
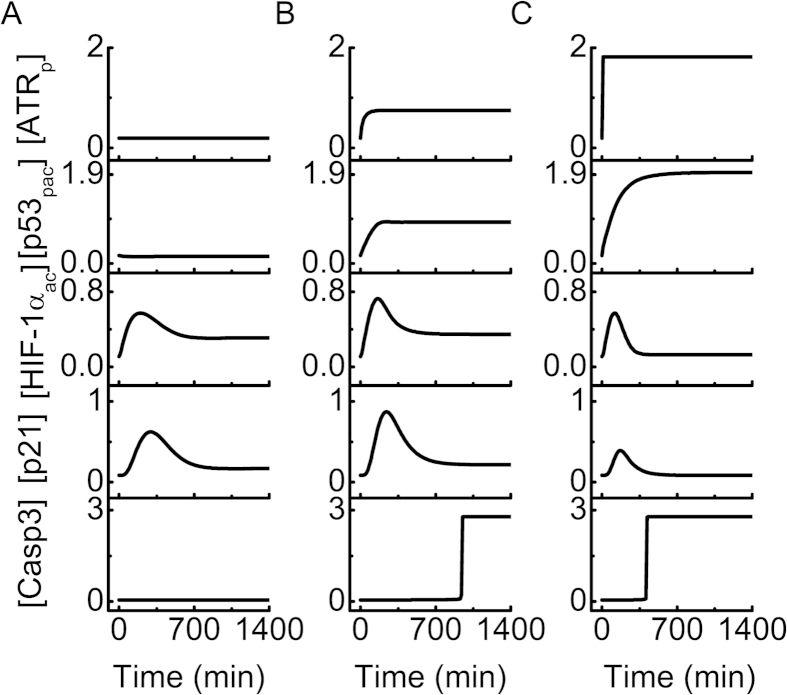
Overview of the network dynamics under different hypoxic conditions. Temporal evolution of the levels of ATR_p_, p53_pac_, HIF-1α_ac_, p21 and Casp3 in mild hypoxia (2% O_2_, (**A**)), severe hypoxia (0.02% O_2_, (**B**)), or anoxia (0% O_2_, (**C**)).

To illustrate the typical dynamics of the HIF-1α and p53 pathways, we present the temporal evolution of protein concentrations under various hypoxic conditions in Fig. 2 ([…] denotes the concentration of each component throughout the paper). In mild hypoxia (2% O_2_), [p53_pac_] is at a basal level because ATR is inactivated, and thus Casp3 remains inactive (Fig. 2A). [HIF-1α_ac_] gradually rises and drops to an intermediate level, since PHD is induced by HIF-1 to promote the degradation of HIF-1α (see Supplemental Fig. S1). p21 is induced by HIF-1 rather than p53 to trigger cell-cycle arrest. Consequently, the cell survives and adapts to mild hypoxia.

Under severe hypoxia (0.02% O_2_), ATR is partly activated, and [p53_pac_] rises to an intermediate level (Fig. 2B). As a result, [HIF-1α_ac_] first rises and then drops to a moderate level since p53_p_ and HIF-1α compete for binding to limiting p300. p53_pac_ represses the expression of miR-17-92 to relieve the inhibition of BIM expression, and [BIM] rises gradually to a relatively high level (see Supplemental Fig. S2). Since [p53_pac_] is not high enough, PUMA only accumulates to a low level. Consequently, Casp3 is activated mainly by BIM around 16 h and apoptosis ensues. These results are consistent with the experimental report that p53 can induce apoptosis via transrepressing antiapoptotic genes^12^,^34^.

Under anoxia (0.0% O_2_), ATR is fully activated and [p53_pac_] rises quickly to a high level (Fig. 2C). Because HIF-1α is degraded by Mdm2 and p300 is mostly associated with p53, [HIF-1α_ac_] returns to its basal level after a transient. Both [BIM] and [PUMA] rise to high levels, and Casp3 is activated around 6 h, which remarkably advances the induction of apoptosis.

The above results indicate that the cellular outcome depends on the extent of hypoxia (note that the hypoxic state is maintained persistently here). In mild hypoxia, [HIF-1α_ac_] settles at an intermediate level, and the cell adapts to the stress after a transient cell-cycle arrest. Under severe hypoxia, the accumulation of p53_pac_ suffices to elevate BIM via downregulating miR-17-92, and apoptosis is induced. In anoxia, the stabilization and transcriptional activity of p53 are markedly enhanced, in sharp contrast to those of HIF-1α. Both transcriptional repression and activation of target genes by p53 are triggered to expedite apoptosis induction, which awaits experimental validation. Notably, the competition for binding to limiting p300 is a key factor modulating the dynamics and activities of p53 and HIF-1α.

### Dynamics of HIF-1α and p21

**Figure 3:**
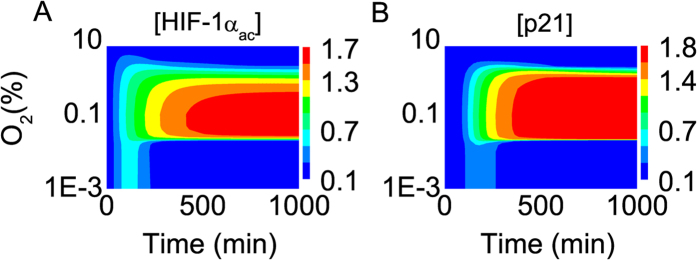
Dynamics of HIF-1α_ac_ and p21. Color-coded concentrations of (**A**) HIF-1α_ac_ and (**B**) p21 as a function of the logarithm of oxygen concentration and time.

To investigate the dynamics of HIF-1α and p21 systematically, we plot the heat maps of [HIF-1α_ac_] and [p21] as a function of oxygen concentration and time (Fig. 3). In normoxia (above 3% O_2_), [HIF-1α_ac_] remains at basal levels since most of HIF-1α is degraded (Fig. 3A). In mild hypoxia (between 1.1% and 3% O_2_), [HIF-1α_ac_] gradually rises and drops due to PHD-dependent degradation. In moderate hypoxia (between 0.03% and 1.1% O_2_), [HIF-1α_ac_] rises to different plateau levels, which can be as high as 1.6. p21 dynamics closely follow the dynamics of HIF-1α_ac_ (Fig. 3B). These results suggest that the cell undergoes a transient cell-cycle arrest in mild hypoxia, or becomes senescent under moderate hypoxia because of sustained p21 induction. It would be interesting to test the occurrence of senescence.

In severe hypoxia (below 0.03% O_2_), HIF-1α first accumulates because of PHD deactivation; then, p53 competes with HIF-1α for binding to p300, leading to a reduction in HIF-1α_ac_ levels and inhibition of HIF-1α activity. In anoxia, p53 competitively binds to p300 and Mdm2 targets HIF-1α for degradation; thus, the HIF-1α_ac_ level drops markedly, and rather low levels of p21 are induced. These results are consistent with the experimental observation that Mdm2 promotes HIF-1α degradation in a p53-dependent way^22^. Together, HIF-1α is partly inactivated under severe hypoxia and is degraded in anoxia.

### p53 dynamics and apoptosis induction under severe hypoxia

**Figure 4:**
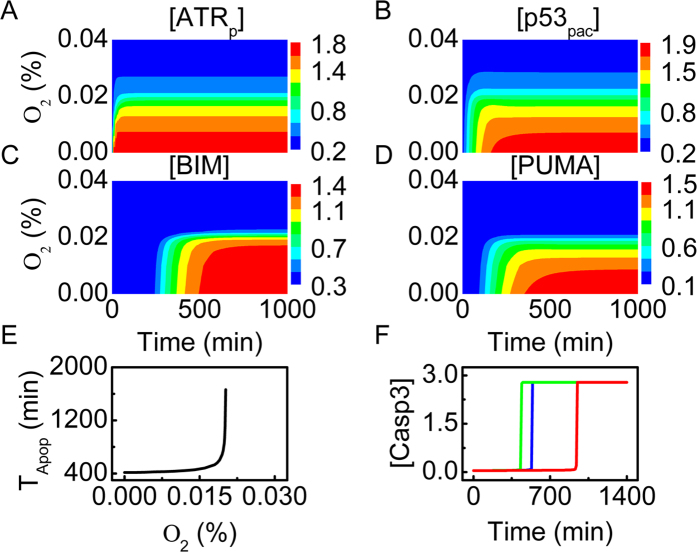
Protein dynamics and apoptosis induction under severe hypoxia. (**A–D**) Color-coded concentrations of ATR_p_ (**A**), p53_pac_ (**B**), BIM (**C**) and PUMA (**D**) as a function of oxygen concentration and time. (**E**) The timing of Casp3 activation versus oxygen concentration. (**F**) Time courses of [Casp3] with oxygen concentration at 0.01% (green), 0.018% (blue), or 0.02% (red).

Under severe hypoxia, ATR is activated, and thus p53 is stabilized by phosphorylation. For oxygen concentration below 0.025%, [ATR_p_] quickly rises to plateau levels (Fig. 4A). ATR is partly activated when oxygen concentration is between 0.01% and 0.025%, or is fully activated when oxygen concentration is below 0.01%. Compared with ATR_p_, p53_pac_ accumulates more slowly. The steady-state level of p53_pac_ rises with decreasing oxygen concentration (Fig. 4B). Of note, we can compare the simulation results with the experimental observations in ref. 8; for comparison, the measured p53 expression level is divided by its maximum (see Supplemental Fig. S3). p53_pac_ accumulates markedly only in severe hypoxia or anoxia, consistent with the experimental result, and the simulation results reproduce the key features of p53 dynamics in response to hypoxia.

Activated p53 represses miR-17-92 expression to upregulate BIM; [BIM] rises to distinct levels depending on oxygen concentration (Fig. 4C). In contrast, only when oxygen concentration is below 0.01% can [PUMA] rise to relatively high levels (Fig. 4D), consistent with the experimental observation^13^. Thus, the transrepression activity of p53 is activated in severe hypoxia, while its transactivation activity is further evoked under anoxia.

Either BIM or PUMA activates Casp3 to trigger apoptosis in hypoxia. The timing of Casp3 activation, *T*_Apop_, versus oxygen concentration is shown in Fig. 4E. Casp3 can be activated around 400 min if oxygen concentration is below 0.015%; otherwise, *T*_Apop_ rises quickly with increasing oxygen concentration. Apoptosis cannot be induced when oxygen concentration is above 0.022%. As seen in Fig. 4F, the timing of Casp3 activation exhibits a significant delay with increasing oxygen concentration. Together, the cell becomes more sensitive to apoptosis with aggravating hypoxia.

### Interplay between HIF-1α and p53 under anoxia

**Figure 5:**
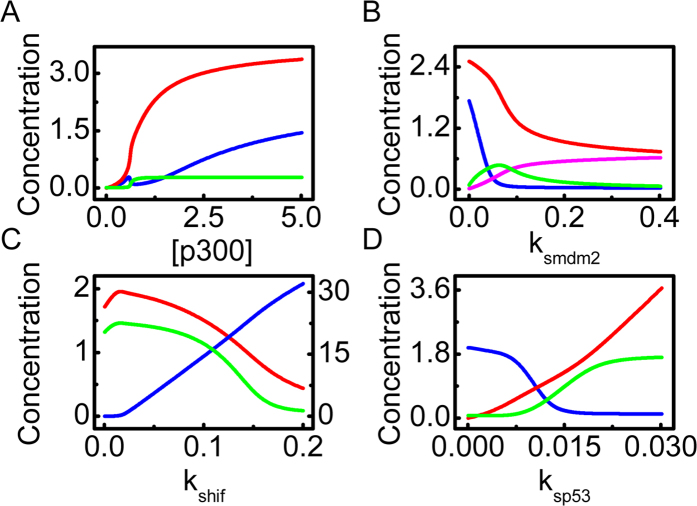
Interplay between HIF-1α and p53 in anoxia. (**A**) Bifurcation diagrams of [p53_pac_] (red), [HIF-1α_ac_] (blue) and [Mdm2_n_] (green) versus the amount of p300. (**B**) Bifurcation diagrams of [p53_pac_] (red), [HIF-1α_ac_] (blue), [Mdm2_c_] (green) and [Mdm2_n_] (pink) versus the p53-induced production rate of Mdm2, *k*_smdm2_. (**C**) Bifurcation diagrams of [HIF-1α_ac_] (blue, on the right axis), [p53_pac_] (red) and [PUMA] (green) versus the production rate of HIF-1α, *k*_shif_. (**D**) Bifurcation diagrams of [HIF-1α_ac_] (blue), [p53_pac_] (red) and [PUMA] (green) versus the production rate of p53, *k*_sp53_.

As mentioned above, p53 and HIF-1α compete for limiting p300 to activate their transcriptional activity and are targeted for degradation by Mdm2. Here, we explore their interplay under anoxia. We first investigate how the p300 level influences the steady-state concentrations of proteins (Fig. 5A). With increasing [p300], [p53_pac_] continuously rises toward saturation. [Mdm2_n_] remains at a low level over a wide range since Mdm2_np_ predominates in the nucleus. Intriguingly, [HIF-1α_ac_] first rises, then drops, and rises again with increasing [p300]. Since ATR is fully activated and [p53_p_] is large enough under anoxia, increasing [p300] leads to enhancement of p53 acetylation. When [p300] is very low, the level of unacetylated HIF-1α is sufficiently high, and [HIF-1α_ac_] rises with increasing [p300]. When [p300] further rises, [p53_pac_] is elevated and HIF-1α is markedly degraded by p53-induced Mdm2, leading to a drop in [HIF-1α_ac_]. If p300 is in excess, more HIF-1α is acetylated, resulting in a continuous rise in [HIF-1α_ac_] with increasing [p300]. The intricate effect of p300 level on the acetylation of HIF-1α is worth testing experimentally.

We further probe the role for Mdm2-mediated degradation of p53 and HIF-1α. With increasing the p53-induced production rate of Mdm2, *k*_smdm2_, [HIF-1α_ac_] first drops fast and then remains at basal levels because of enhanced degradation of HIF-1α by Mdm2 (Fig. 5B). In contrast, [p53_pac_] falls at a slower rate and always remains above an intermediate level. At large *k*_smdm2_, Mdm2_n_ predominates over Mdm2_c_ since [p53_pac_] is not sufficiently high to transrepress miR-17-92 markedly, which promotes the nuclear entry of Mdm2. Collectively, Mdm2 overexpression inhibits the transcriptional activities of both HIF-1 and p53.

The expression level of HIF-1α has a marked effect on the activities of HIF-1α and p53. The amount of HIF-1α depends on its production rate *k*_shif_. For very low *k*_shif_, [p53_pac_] remains at high levels, whereas there is little amount of HIF-1α_ac_ (Fig. 5C). When *k*_shif_ is further increased, more HIF-1α is produced and [HIF-1α_ac_] gradually rises, leading to a drop in [p53_pac_] because of their competition for limiting p300. Since PUMA expression is p53-dependent, [PUMA] shows the similar dynamics to [p53_pac_]. Thus, p53 cannot be activated to induce apoptosis even under anoxia when HIF-1α is overexpressed.

Similarly, the p53 expression level affects the activities of HIF-1α and p53. With increasing the rate constant of p53 production, *k*_sp53_, [p53_pac_] rises continuously, whereas [HIF-1α_ac_] drops to basal levels (Fig. 5D). As a result, [PUMA] gradually rises toward saturation at large *k*_sp53_. Of note, [HIF-1α_ac_] remains at high levels with *k*_sp53_=0, and apoptosis cannot be induced even in anoxia. This result is consistent with the fact that cells with p53 mutation are resistant to proapoptotic stimuli^38^. Together, the competition between p53 and HIF-1α for p300 and Mdm2 plays a key role in the modulation of their stabilization and transcriptional activity.

### Effect of miR-17-92 repression on apoptosis induction

In addition to repression of FOXO-induced BIM expression, miR-17-92 also indirectly suppresses FOXO and p53 activities by inhibiting PTEN expression^30^. Here, we clarify the significance of miR-17-92 inhibition by p53 in hypoxia-induced apoptosis. Note that the parameter *j*_smir_ characterizes the repression strength by p53: the larger *j*_smir_, the weaker the repression becomes (see Eq. 21).

**Figure 6:**
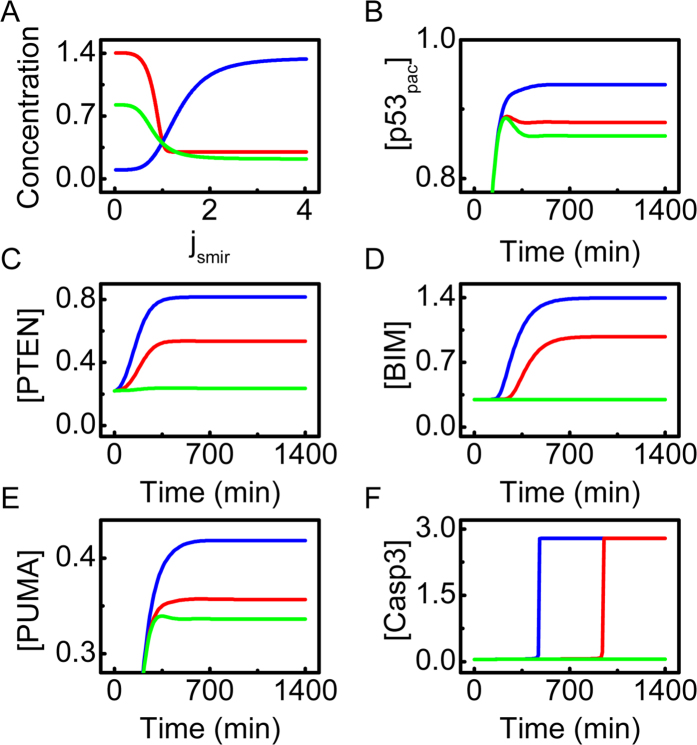
Effect of miR-17-92 on apoptosis induction under severe hypoxia (0.02% O_2_). (**A**) Bifurcation diagrams of [PTEN] (green), [BIM] (red) and [miR-17-92] (blue) as a function of *j*_smir_. (**B**–**F**) Time courses of [p53_pac_] (**B**), [PTEN] (**C**), [BIM] (**D**), [PUMA] (**E**) and [Casp3] (**F**) with *j*_smir_ = 0.3 (blue), 0.8 (red), or 2.0 (green).

As shown in the bifurcation diagrams, [PTEN] and [BIM] remain at relatively high levels for *j*_smir  _≤ 1, whereas [miR-17-92] is at low levels (Fig. 6A). In contrast, [PTEN] and [BIM] stay at low levels once *j*_smir _> 1, whereas [miR-17-92] gradually rises with increasing *j*_smir_. This suggests that the effective transrepression of miR-17-92 by p53 is indispensable for apoptosis induction under severe hypoxia, which awaits experimental validation.

We further show the temporal evolution of protein concentrations for different values of *j*_smir_ in Fig. 4B–F. At *j*_smir_ = 0.3, [PTEN] reaches a high level, promoting the accumulation of p53. High expression of BIM and PUMA activates Casp3 to trigger apoptosis promptly. If *j*_smir_ is increased to 0.8, the expression of PTEN and BIM is reduced, and the levels of p53_pac_ and PUMA are downregulated. Consequently, Casp3 is activated with a marked delay. At *j*_smir_ = 2.0, the repression of miR-17-92 expression by p53 becomes very weak, and apoptosis cannot be induced. Collectively, the transrepression of miR-17-92 is significant for apoptosis induction under severe hypoxia.

## Discussion

In this work, we provide a coherent picture of cell-fate decision under hypoxic conditions. Under normoxia, both HIF-1α and p53 are at very low levels. In mild hypoxia, p53 still remains inactive, whereas HIF-1α first accumulates to induce transient cell-cycle arrest and then settles at moderate levels because of the cellular adaptation to hypoxia. Under moderate hypoxia, HIF-1α_ac_ expression is sufficiently high, and p21 induction is maintained to trigger cellular senescence. In severe hypoxia, p53 accumulation attenuates HIF-1α activity, and p53 transrepresses antiapoptotic genes like miR-17-92 to relieve inhibition of proapoptotic genes like BIM, thereby inducing apoptosis. Under anoxia, high levels of p53 lead to HIF-1α degradation, and p53 transrepresses and transactivates target genes to remarkably advance apoptosis induction. Such a decision mechanism seems plausible, either promoting cellular adaptation to mild stress or inducing cell death after severe stress.

The interplay between p53 and HIF-1α plays a key role in the hypoxic response. Our results are consistent with experimental observations: moderate p53 expression attenuates HIF-1α activity, whereas high p53 expression eliminates HIF-1α activity^39^. Notably, HIF-1α is overexpressed in most cancers^40^, and thus developing HIF-1α inhibitors to prevent the adaptation of tumor cells to hypoxia may be a promising strategy for anticancer^41^. Our results suggest that reactivation of p53 could contribute to inhibition of HIF-1α activity.

The mechanism for p53 stabilization is somehow controversial in the literature. Previously, it was reported that p53 was stabilized by HIF-1α^16^. Later, this mechanism was challenged by the report that p53 accumulated only in severe hypoxia^8^. Thus, HIF-1α alone cannot stabilize p53. Rather, it was found that ATR contributes to p53 stabilization in severe hypoxia when replication arrest occurs. Here, we further show that the competition between p53 and HIF-1α for limiting p300 not only determines their transcriptional activity, but also regulates their abundance because p300-mediated acetylation prevents Mdm2-mediated protein degradation. p53 stabilization is enhanced when more Mdm2 is associated with unacetylated HIF-1α.

Our work may reconcile the contradictory reports on the mechanism for hypoxia-induced apoptosis^11^,^12^,^13^. We propose that depending on the severity of hypoxia, p53 may suppress the expression of antiapoptotic genes like miR-17-92, or inhibit miR-17-92 expression and activate PUMA expression synergistically to induce apoptosis. Accordingly, it takes a relatively long time to evoke apoptosis under severe hypoxia, or apoptosis is induced promptly after exposure to anoxia. Such an action mode of p53 is of functional significance, allowing for cellular adaptation to the hypoxic environment or eliminating cells promptly under strongly anoxic conditions. It would be interesting to experimentally test that.

We showed that effective repression of miR-17-92 expression is required for apoptosis induction under severe hypoxia. miR-17-92 itself can inhibit the production of BIM and PTEN^34^. Conversely, p53 transrepresses miR-17-92 to elevate BIM and PTEN expression^12^. BIM can induce apoptosis^42^, while PTEN contributes to p53 stabilization by sequestering Mdm2 in the cytoplasm^32^. It is worthy to explore whether other microRNAs induced by p53, such as miR-34a, contribute to apoptosis induction in hypoxia.

Finally, it is worth noting that the cellular response to hypoxia is extremely complicated. Here, we took a simplified approach to characterize the crosstalk between HIF-1α and p53 signaling by focusing on their competition in stabilization and transcriptional activity. We probed the effect of the severity of hypoxia on cell fate, while omitting the influence of the duration of hypoxia. Some other factors were also ignored, such as complex relationships between HIF-1, p53 and pVHL^7^ and different phosphorylation modifications of p53. Definitely, adding more facets of signal transduction to the model should shed new light on the hypoxic response.

## Methods

The details of the model are presented in the Supporting Material. The concentration of each component is represented by a dimensionless state variable. The temporal evolution of dynamic systems is governed by ordinary differential equations, which are presented in Supplemental Method. All the initial values of variables are their lower steady-state values under normoxia and are listed in Supplemental Table S1. The standard parameter values are listed in Supplemental Table S2. Some parameters are set based on experimental measurements or known facts, while others are estimated by comparing the simulation results with experimental data. The unit of time is minutes, while the units of parameters are determined such that the concentrations of proteins are dimensionless. For simplicity, we do not indicate the units of the parameters explicitly. The rate equations are numerically solved using Oscill8.

The robustness of protein concentrations to parameter variations is also analyzed, and the results are presented in Supplemental Table S3. The sensitivity of steady-state levels of 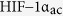 and p53_pac_ to parameter variations is calculated when the parameter values are varied by ±10% with respect to their standard values. These concentrations are only sensitive to the parameters related to HIF-1α regulation, Mdm2-mediated protein degradation, or p53 production. Overall, our simulation results are robust to changes in most parameters, and we provide a model for effectively exploring the interplay between p53 and 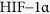 in response to hypoxia.

## Additional Information

**How to cite this article**: Zhou, C.-H. *et al.* Modeling the interplay between the HIF-1 and p53 pathways in hypoxia. *Sci. Rep.* **5**, 13834; doi: 10.1038/srep13834 (2015).

## Supplementary Material

Supplementary Information

## Figures and Tables

**Figure 1:**
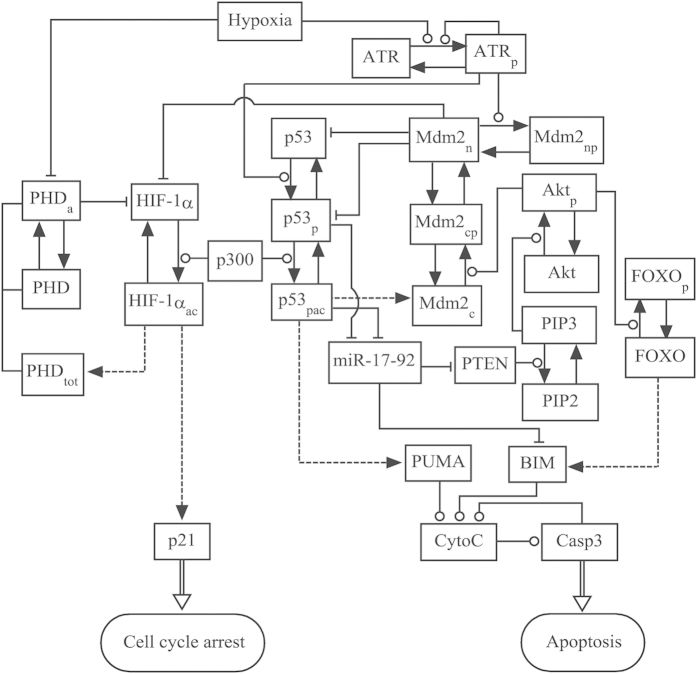
Schematic depiction of the model. The model characterizes crosstalk between the HIF-1 and p53 pathways upon hypoxia. In hypoxia, HIF-1α is stabilized due to reduced hydroxylation by PHD. Under severe hypoxia, the ATR kinase is activated via auto-phosphorylation upon hypoxia-induced replication arrest, and p53 is further activated by ATR. The shared coactivator p300 is required for the full transcriptional activity of p53 and HIF-1α. HIF-1α evokes transient cell-cycle arrest via inducing p21, whereas p53 can induce apoptosis via transrepressing or/and transactivating target genes. Dashed lines denote the expression of target genes by HIF-1α or p53, while solid arrowed lines represent the transitions between proteins. Circle- and bar-headed lines denote the promotion and inhibition of transition or production, respectively.

## References

[b1] Semenza G. L. HIF-1: Upstream and downstream of cancer metabolism. Curr. Opin. Genet. Dev. 20, 51–56 (2010). 19942427 10.1016/j.gde.2009.10.009 PMC2822127

[b2] Wang G. L., Jiang B. H., Rue E. A. & Semenza G. L. Hypoxia-inducible factor 1 is a basic-helix-loop-helix-PAS heterodimer regulated by cellular O_2_ tension. Proc. Natl. Acad. Sci. USA 92, 5510–5514 (1995). 7539918 10.1073/pnas.92.12.5510 PMC41725

[b3] Ivan M. *et al.* HIFα targeted for VHL-mediated destruction by proline hydroxylation: Implications for O_2_ sensing. Science 292, 464–468 (2001). 11292862 10.1126/science.1059817

[b4] Lando D. *et al.* FIH-1 is an asparaginyl hydroxylase enzyme that regulates the transcriptional activity of hypoxia-inducible factor. Genes Dev. 16, 1466–1471 (2002). 12080085 10.1101/gad.991402 PMC186346

[b5] Semenza G. L. Targeting HIF-1 for cancer therapy. Nat. Rev. Cancer 3, 721–732 (2003). 13130303 10.1038/nrc1187

[b6] Greer S. N., Metcalf J. L., Wang Y. & Ohh M. The updated biology of hypoxia-inducible factor. EMBO J. 31, 2448–2460 (2012). 22562152 10.1038/emboj.2012.125 PMC3365421

[b7] Sermeus A. & Michiels C. Reciprocal influence of the p53 and the hypoxic pathways. Cell Death Dis. 2, e164 (2011). 21614094 10.1038/cddis.2011.48 PMC3122125

[b8] Hammond E. M., Denko N. C., Dorie M. J., Abraham R. T. & Giaccia A. J. Hypoxia links ATR and p53 through replication arrest. Mol. Cell. Biol. 22, 1834–1843 (2002). 11865061 10.1128/MCB.22.6.1834-1843.2002 PMC135616

[b9] Zhang X. P., Liu F. & Wang W. Two-phase dynamics of p53 in the DNA damage response. Proc. Natl. Acad. Sci. USA 108, 8990–8995 (2011). 21576488 10.1073/pnas.1100600108 PMC3107314

[b10] Fridman J. S. & Lowe S. W. Control of apoptosis by p53. Oncogene 22, 9030–9040 (2003). 14663481 10.1038/sj.onc.1207116

[b11] Koumenis C. *et al.* Regulation of p53 by hypoxia: Dissociation of transcriptional repression and apoptosis from p53-dependent transactivation. Mol. Cell. Biol. 21, 1297–1310 (2001). 11158315 10.1128/MCB.21.4.1297-1310.2001 PMC99582

[b12] Yan H. L. *et al.* Repression of the miR-17-92 cluster by p53 has an important function in hypoxia-induced apoptosis. EMBO J. 28, 2719–2732 (2009). 19696742 10.1038/emboj.2009.214 PMC2750010

[b13] Yu J., Wang Z., Kinzler K. W., Vogelstein B. & Zhang L. PUMA mediates the apoptotic response to p53 in colorectal cancer cells. Proc. Natl. Acad. Sci. USA 100, 1931–1936 (2003). 12574499 10.1073/pnas.2627984100 PMC149936

[b14] Liu T. *et al.* Hypoxia induces p53-dependent transactivation and Fas/CD95-dependent apoptosis. Cell Death Differ. 14, 411–421 (2006). 16917513 10.1038/sj.cdd.4402022

[b15] Fei P. *et al.* Bnip3L is induced by p53 under hypoxia, and its knockdown promotes tumor growth. Cancer Cell 6, 597–609 (2004). 15607964 10.1016/j.ccr.2004.10.012

[b16] An W. G. *et al.* Stabilization of wild-type p53 by hypoxia-inducible factor 1α. Nature 392, 405–408 (1998). 9537326 10.1038/32925

[b17] Chen D., Li M., Luo J. & Gu W. Direct interactions between HIF-1α and Mdm2 modulate p53 function. J. Biol. Chem. 278, 13595–13598 (2003). 12606552 10.1074/jbc.C200694200

[b18] Schmid T., Zhou J., Köhl R. & Brüne B. p300 relieves p53-evoked transcriptional repression of hypoxia-inducible factor-1 (HIF-1). Biochem. J. 380, 289–295 (2004). 14992692 10.1042/BJ20031299 PMC1224165

[b19] Qutub A. A. & Popel A. S. A computational model of intracellular oxygen sensing by hypoxia-inducible factor HIF1α. J. Cell Sci. 119, 3467–3480 (2006). 16899821 10.1242/jcs.03087 PMC2129128

[b20] Schmierer B., Novák B. & Schofield C. J. Hypoxia-dependent sequestration of an oxygen sensor by a widespread structural motif can shape the hypoxic response - a predictive kinetic model. BMC Syst. Biol. 4, 139 (2010). 20955552 10.1186/1752-0509-4-139 PMC2984394

[b21] Nguyen L. K. *et al.* A dynamic model of the hypoxia-inducible factor 1α (HIF-1α) network. J. Cell Sci. 126, 1454–1463 (2013). 23390316 10.1242/jcs.119974

[b22] Ravi R. *et al.* Regulation of tumor angiogenesis by p53-induced degradation of hypoxia-inducible factor 1α. Genes Dev. 14, 34–44 (2000). 10640274 PMC316350

[b23] Arany Z. *et al.* An essential role for p300/CBP in the cellular response to hypoxia. Proc. Natl. Acad. Sci. USA 93, 12969–12973 (1996). 8917528 10.1073/pnas.93.23.12969 PMC24030

[b24] Avantaggiati M. L. *et al.* Recruitment of p300/CBP in p53-dependent signal pathways. Cell 89, 1175–1184 (1997). 9215639 10.1016/s0092-8674(00)80304-9

[b25] D’Angelo G., Duplan E., Boyer N., Vigne P. & Frelin C. Hypoxia up-regulates prolyl hydroxylase activity: A feedback mechanism that limits HIF-1 responses during reoxygenation. J. Biol. Chem. 278, 38183–38187 (2003). 12876291 10.1074/jbc.M302244200

[b26] Stiehl D. P. *et al.* Increased prolyl 4-hydroxylase domain proteins compensate for decreased oxygen levels: Evidence for an autoregulatory oxygen-sensing system. J. Biol. Chem. 281, 23482–23491 (2006). 16790428 10.1074/jbc.M601719200

[b27] Liu S. *et al.* ATR autophosphorylation as a molecular switch for checkpoint activation. Mol. Cell 43, 192–202 (2011). 21777809 10.1016/j.molcel.2011.06.019 PMC3155885

[b28] Kholodenko B. N. Cell-signalling dynamics in time and space. Nat. Rev. Mol. Cell Biol. 7, 165–176 (2006). 16482094 10.1038/nrm1838 PMC1679905

[b29] Hammond E. M., Dorie M. J. & Giaccia A. J. ATR/ATM targets are phosphorylated by ATR in response to hypoxia and ATM in response to reoxygenation. J. Biol. Chem. 278, 12207–12213 (2003). 12519769 10.1074/jbc.M212360200

[b30] Shinozaki T., Nota A., Taya A. & Okamoto K. Functional role of Mdm2 phosphorylation by ATR in attenuation of p53 nuclear export. Oncogene 22, 8870–8880 (2003). 14654783 10.1038/sj.onc.1207176

[b31] Mayo L. D. & Donner D. B. A phosphatidylinositol 3-kinase/Akt pathway promotes translocation of Mdm2 from the cytoplasm to the nucleus. Proc. Natl. Acad. Sci. USA 98, 11598–11603 (2001). 11504915 10.1073/pnas.181181198 PMC58775

[b32] Manning B. D. & Cantley L. C. AKT/PKB signaling: Navigating downstream. Cell 129, 1261–1274 (2007). 17604717 10.1016/j.cell.2007.06.009 PMC2756685

[b33] Wee K. B. & Aguda B. D. Akt versus p53 in a network of oncogenes and tumor suppressor genes regulating cell survival and death. Biophys. J. 91, 857–865 (2006). 16648169 10.1529/biophysj.105.077693 PMC1563780

[b34] Xiao C. *et al.* Lymphoproliferative disease and autoimmunity in mice with increased miR-17-92 expression in lymphocytes. Nat. Immunol. 9, 405–414 (2008). 18327259 10.1038/ni1575 PMC2533767

[b35] Kloet D. E. A. & Burgering B. M. T. The PKB/FOXO switch in aging and cancer. BBA-Mol. Cell Res. 1813, 1926–1937 (2011). 10.1016/j.bbamcr.2011.04.003 21539865

[b36] Cory S. & Adams J. M. The Bcl2 family: Regulators of the cellular life-or-death switch. Nat. Rev. Cancer 2, 647–56 (2002). 12209154 10.1038/nrc883

[b37] Kirsch D. G. *et al.* Caspase-3-dependent cleavage of Bcl-2 promotes release of cytochrome c. J. Biol. Chem. 274, 21155–21161 (1999). 10409669 10.1074/jbc.274.30.21155

[b38] Levine A. J. p53, the cellular gatekeeper for growth and division. Cell 88, 323–331 (1997). 9039259 10.1016/s0092-8674(00)81871-1

[b39] Schofield C. J. & Ratcliffe P. J. Oxygen sensing by HIF hydroxylases. Nat. Rev. Mol. Cell Biol. 5, 343–354 (2004). 15122348 10.1038/nrm1366

[b40] Talks K. L. *et al.* The expression and distribution of the hypoxia-inducible factors HIF-1α and HIF-2α in normal human tissues, cancers, and tumor-associated macrophages. Am. J. Pathol. 157, 411–421 (2000). 10934146 10.1016/s0002-9440(10)64554-3 PMC1850121

[b41] Xia Y., Choi H. K. & Lee K. Recent advances in hypoxia-inducible factor (HIF)-1 inhibitors. Eur. J. Med. Chem. 49, 24–40 (2012). 22305612 10.1016/j.ejmech.2012.01.033

[b42] Bouillet P. *et al.* Proapoptotic Bcl-2 relative Bim required for certain apoptotic responses, leukocyte homeostasis, and to preclude autoimmunity. Science 286, 1735–1738 (1999). 10576740 10.1126/science.286.5445.1735

