# Supplementary material for: Modeling the interplay between the HIF-1 and p53 pathways in hypoxia
Source: Sci Rep. 2015 Sep 8;5:13834. doi: 10.1038/srep13834 (PMC4561886; doi:10.1038/srep13834)
Supplement: Supplementary Information [file srep13834-s1.pdf]

SUPPORTING MATERIAL

Modeling the interplay between the HIF-1 and p53 pathways in hypoxia

Chun-Hong Zhou, Xiao-Peng Zhang, Feng Liu, and Wei Wang

In this work, we mainly explored the crosstalk between the HIF-1 and p53 pathways in response to hypoxia. We built a network model, characterizing the activation of HIF-1α and p53, selective expression of target genes, and apoptosis induction. This document includes the ordinary differential equations for the model, three tables, and three figures.

Supplemental Method: Equations of the model

$$
\begin{aligned}
\frac{d[\text{HIF-1}\alpha]}{dt} &= k_{\text{shif}} - k_{\text{dhif}}[\text{HIF-1}\alpha] - k_{\text{dhifo}_2}[\text{PHD}_\text{a}]\frac{[\text{HIF-1}\alpha]}{[\text{HIF-1}\alpha]+j_{\text{dhifo}_2}} \\
&\quad - k_{\text{achif}}[\text{p300}]\frac{[\text{HIF-1}\alpha]}{[\text{HIF-1}\alpha]+[\text{p53}_\text{p}]\frac{j_{\text{achif}}}{j_{\text{acp53}}}+j_{\text{achif}}} + k_{\text{dachif}}\frac{[\text{HIF-1}\alpha_{\text{ac}}]}{[\text{HIF-1}\alpha_{\text{ac}}]+j_{\text{dachif}}} \\
&\quad - k_{\text{dmhif}}[\text{Mdm2}_\text{n}]\frac{[\text{HIF-1}\alpha]}{[\text{HIF-1}\alpha]+[\text{p53}]\frac{j_{\text{dmhif}}}{j_{\text{dmp53}}}+[\text{p53}_\text{p}]\frac{j_{\text{dmhif}}}{j_{\text{dmp53p}}}+j_{\text{dmhif}}} &(1)\\
\frac{d[\text{HIF-1}\alpha_{\text{ac}}]}{dt} &= k_{\text{achif}}[\text{p300}]\frac{[\text{HIF-1}\alpha]}{[\text{HIF-1}\alpha]+[\text{p53}_\text{p}]\frac{j_{\text{achif}}}{j_{\text{acp53}}}+j_{\text{achif}}} - k_{\text{dachif}}\frac{[\text{HIF-1}\alpha_{\text{ac}}]}{[\text{HIF-1}\alpha_{\text{ac}}]+j_{\text{dachif}}} \\
&\quad - k_{\text{dhifac}}[\text{HIF-1}\alpha_{\text{ac}}] &(2)\\
\frac{d[\text{p21}]}{dt} &= k_{\text{sp210}} + k_{\text{sp21}}\frac{[\text{HIF-1}\alpha_{\text{ac}}]^4}{[\text{HIF-1}\alpha_{\text{ac}}]^4+j_{\text{sp21}}^4} - k_{\text{dp21}}[\text{p21}] &(3)\\
\frac{d[\text{PHD}_{\text{tot}}]}{dt} &= k_{\text{sphd0}} + k_{\text{sphd}}\frac{[\text{HIF-1}\alpha_{\text{ac}}]^4}{[\text{HIF-1}\alpha_{\text{ac}}]^4+j_{\text{sphd}}^4} - k_{\text{dphd}}[\text{PHD}_{\text{tot}}] &(4)\\
[\text{PHD}] &= \text{PHD}_{\text{tot}} - [\text{PHD}_\text{a}] &(5)\\
\frac{d[\text{PHD}_\text{a}]}{dt} &= k_{\text{acphd}}\frac{o_2}{o_2+j_{o_2}}\frac{[\text{PHD}]}{[\text{PHD}]+j_{\text{acphd}}} - k_{\text{dephd}}\frac{[\text{PHD}_\text{a}]}{[\text{PHD}_\text{a}]+j_{\text{dephd}}} &(6)\\
[\text{ATR}] &= \text{ATR}_{\text{tot}} - [\text{ATR}_\text{p}] &(7)\\
\frac{d[\text{ATR}_\text{p}]}{dt} &= \left(k_{\text{acatr0}} + k_{\text{acatr}}\frac{k_{o_2}}{o_2+k_{o_2}}[\text{ATR}_\text{p}]\right)\frac{[\text{ATR}]}{[\text{ATR}]+j_{\text{acatr}}} - k_{\text{deatr}}\frac{[\text{ATR}_\text{p}]}{[\text{ATR}_\text{p}]+j_{\text{deatr}}} &(8)\\
\frac{d[\text{p53}]}{dt} &= k_{\text{sp53}} - k_{\text{dp53}}[\text{p53}] + k_{\text{dpp53}}\frac{[\text{p53}_\text{p}]}{[\text{p53}_\text{p}]+j_{\text{dpp53}}} - k_{\text{pp53}}[\text{ATR}_\text{p}]\frac{[\text{p53}]}{[\text{p53}]+j_{\text{pp53}}} \\
&\quad - k_{\text{dmp53}}[\text{Mdm2}_\text{n}]\frac{[\text{p53}]}{[\text{HIF-1}\alpha]\frac{j_{\text{dmp53}}}{j_{\text{dmhif}}}+[\text{p53}]+[\text{p53}_\text{p}]\frac{j_{\text{dmp53}}}{j_{\text{dmp53p}}}+j_{\text{dmp53}}} &(9)
\end{aligned}
$$

$$\frac{d[\text{p53}_\text{p}]}{dt} = k_\text{pp53}[\text{ATR}_\text{p}]\frac{[\text{p53}]}{[\text{p53}]+j_\text{pp53}} - k_\text{dpp53}\frac{[\text{p53}_\text{p}]}{[\text{p53}_\text{p}]+j_\text{dpp53}} - k_\text{dp53p}[\text{p53}_\text{p}]$$
$$-k_\text{acp53}[\text{p300}]\frac{[\text{p53}_\text{p}]}{[\text{HIF-1}\alpha]\frac{j_\text{acp53}}{j_\text{achif}}+[\text{p53}_\text{p}]+j_\text{acp53}} + k_\text{dacp53}\frac{[\text{p53}_\text{pac}]}{[\text{p53}_\text{pac}]+j_\text{dacp53}}$$
$$-k_\text{dmp53p}[\text{Mdm2}_\text{n}]\frac{[\text{p53}_\text{p}]}{[\text{HIF-1}\alpha]\frac{j_\text{dmp53p}}{j_\text{dmhif}}+[\text{p53}_\text{p}]+[\text{p53}]\frac{j_\text{dmp53p}}{j_\text{dmp53}}+j_\text{dmp53p}} \quad (10)$$

$$\frac{d[\text{p53}_\text{pac}]}{dt} = k_\text{acp53}[\text{p300}]\frac{[\text{p53}_\text{p}]}{[\text{HIF-1}\alpha]\frac{j_\text{acp53}}{j_\text{achif}}+[\text{p53}_\text{p}]+j_\text{acp53}} - k_\text{dacp53}\frac{[\text{p53}_\text{pac}]}{[\text{p53}_\text{pac}]+j_\text{dacp53}}$$
$$-k_\text{dp53pac}[\text{p53}_\text{pac}] \quad (11)$$

$$\frac{d[\text{Mdm2}_\text{c}]}{dt} = k_\text{smdm20} + k_\text{smdm2}\frac{[\text{p53}_\text{pac}]^4}{[\text{p53}_\text{pac}]^4+j_\text{smdm2}^4} - k_\text{dmdm2}[\text{Mdm2}_\text{c}]$$
$$-k_\text{mdm2s}[\text{Akt}_\text{p}]\frac{[\text{Mdm2}_\text{c}]}{[\text{Mdm2}_\text{c}]+j_\text{mdm2s}} + k_\text{1mdm2s}\frac{[\text{Mdm2}_\text{cp}]}{[\text{Mdm2}_\text{cp}]+j_\text{1mdm2s}} \quad (12)$$

$$\frac{d[\text{Mdm2}_\text{cp}]}{dt} = k_\text{mdm2s}[\text{Akt}_\text{p}]\frac{[\text{Mdm2}_\text{c}]}{[\text{Mdm2}_\text{c}]+j_\text{mdm2s}} - k_\text{1mdm2s}\frac{[\text{Mdm2}_\text{cp}]}{[\text{Mdm2}_\text{cp}]+j_\text{1mdm2s}}$$
$$-k_\text{i}[\text{Mdm2}_\text{cp}] + k_\text{o}[\text{Mdm2}_\text{n}] - k_\text{dmdm2}[\text{Mdm2}_\text{cp}] \quad (13)$$

$$\frac{d[\text{Mdm2}_\text{n}]}{dt} = k_\text{i}[\text{Mdm2}_\text{cp}] - k_\text{o}[\text{Mdm2}_\text{n}] - k_\text{pmdm2}[\text{ATR}_\text{p}]\frac{[\text{Mdm2}_\text{n}]}{[\text{Mdm2}_\text{n}]+j_\text{pmdm2}}$$
$$+k_\text{dpmdm2}\frac{[\text{Mdm2}_\text{np}]}{[\text{Mdm2}_\text{np}]+j_\text{dpmdm2}} - k_\text{dmdm2n}[\text{Mdm2}_\text{n}] \quad (14)$$

$$\frac{d[\text{Mdm2}_\text{np}]}{dt} = k_\text{pmdm2}[\text{ATR}_\text{p}]\frac{[\text{Mdm2}_\text{n}]}{[\text{Mdm2}_\text{n}]+j_\text{pmdm2}}$$
$$-k_\text{dpmdm2}\frac{[\text{Mdm2}_\text{np}]}{[\text{Mdm2}_\text{np}]+j_\text{dpmdm2}} - k_\text{dmdm2n}[\text{Mdm2}_\text{np}] \quad (15)$$

$$[\text{Akt}] = \text{Akt}_\text{tot} - [\text{Akt}_\text{p}] \quad (16)$$

$$\frac{d[\text{Akt}_\text{p}]}{dt} = k_\text{acakt}[\text{PIP3}]\frac{[\text{Akt}]}{[\text{Akt}]+j_\text{acakt}} - k_\text{deakt}\frac{[\text{Akt}_\text{p}]}{[\text{Akt}_\text{p}]+j_\text{deakt}} \quad (17)$$

$$[\text{PIP2}] = \text{PIP}_\text{tot} - [\text{PIP3}] \quad (18)$$

$$\frac{d[\text{PIP3}]}{dt} = k_\text{p2}\frac{[\text{PIP2}]}{[\text{PIP2}]+j_\text{p2}} - k_\text{p3}[\text{PTEN}]\frac{[\text{PIP3}]}{[\text{PIP3}]+j_\text{p3}} \quad (19)$$

$$\frac{d[\text{PUMA}]}{dt} = k_\text{spuma0} + k_\text{spuma}\frac{[\text{p53}_\text{pac}]^4}{[\text{p53}_\text{pac}]^4+j_\text{spuma}^4} - k_\text{dpuma}[\text{PUMA}] \quad (20)$$

$$\frac{d[\text{miR-17-92}]}{dt} = k_\text{s0mir17} + k_\text{smir17}\frac{j_\text{smir}^4}{([\text{p53}_\text{p}]+[\text{p53}_\text{pac}])^4+j_\text{smir}^4} - k_\text{dmir17}[\text{miR-17-92}] \quad (21)$$

$$\frac{d[\text{PTEN}]}{dt} = k_\text{spten0} + k_\text{spten}\frac{j_\text{spten}}{[\text{miR-17-92}]+j_\text{spten}} - k_\text{dpten}[\text{PTEN}] \quad (22)$$

$$\frac{d[\text{BIM}]}{dt} = k_\text{sbim0} + k_\text{sbim}\frac{[\text{FOXO}]^4}{[\text{FOXO}]^4+j_\text{sbim}^4}\frac{j_\text{smbim}}{[\text{miR-17-92}]+j_\text{smbim}} - k_\text{dbim}[\text{BIM}] \quad (23)$$

$$
\begin{aligned}
[\mathrm{FOXO_p}] &= \mathrm{FOXO_{tot}} - [\mathrm{FOXO}] & (24)\\
\frac{d[\mathrm{FOXO}]}{dt} &= k_{\mathrm{dpfoxo}}\frac{[\mathrm{FOXO_p}]}{[\mathrm{FOXO_p}] + j_{\mathrm{dpfoxo}}} - k_{\mathrm{acfoxo}}[\mathrm{Akt_p}]\frac{[\mathrm{FOXO}]}{[\mathrm{FOXO}] + j_{\mathrm{acfoxo}}} & (25)\\
\frac{d[\mathrm{Casp3}]}{dt} &= (k_{\mathrm{acasp30}} + k_{\mathrm{acasp31}}\frac{[\mathrm{CytoC}]^4}{[\mathrm{CytoC}]^4 + j_{\mathrm{cytoc}}^4})(\mathrm{Casp3_{tot}} - [\mathrm{Casp3}]) & \\
&\quad -k_{\mathrm{decasp3}}[\mathrm{Casp3}] & (26)\\
\frac{d[\mathrm{CytoC}]}{dt} &= [k_{\mathrm{accytoc0}} + (k_{\mathrm{puma}}[\mathrm{PUMA}] + k_{\mathrm{bim}}[\mathrm{BIM}])\frac{[\mathrm{Casp3}]^4}{[\mathrm{Casp3}]^4 + j_{\mathrm{casp3}}^4}] & \\
&\quad (\mathrm{CytoC_{tot}} - [\mathrm{CytoC}])\frac{j_{\mathrm{p21c}}}{[\mathrm{p21}] + j_{\mathrm{p21c}}} - k_{\mathrm{decytoC}}[\mathrm{CytoC}] & (27)
\end{aligned}
$$

Robustness analysis of the model

We analyze the robustness of HIF-1α and p53 dynamics to parameter variations. Although there exist 106 parameters altogether in the model, we only select 26 parameters involved in HIF-1α and p53 regulation since we focus on the interplay between HIF-1α and p53. The steady-state levels of HIF-1α_{ac} and $\mathrm{p53_{pac}}$ in the cellular response to anoxia are chosen to describe the properties of HIF-1α and p53 dynamics. Each parameter is increased or decreased by 10% with respect to the default setting, and the relative changes of [HIF-1α_{ac}] and [$\mathrm{p53_{pac}}$], L_{HIF}% and L_{p53}%, are listed in Table S3.

HIF-1α_{ac} and $\mathrm{p53_{pac}}$ exhibit different sensitivity to parameter variations. [HIF-1α_{ac}] is more sensitive to the following parameters: the parameters related to HIF-1α regulation (k_{shif0}, k_{dahif}, k_{achif}, j_{achif}) and those related to Mdm2-mediated degradation (k_{pmdm2}, k_{smdm2}, k_{spten}, k_{dpten}). [$\mathrm{p53_{pac}}$] is most sensitive to the basal synthesis rate of p53 (k_{sp53}). Overall, our results are fairly robust to changes in most parameters.

SUPPLEMENTAL TABLE S1: DESCRIPTION AND INITIAL VALUES OF VARIABLES

Variable	Description	Initial value
$[\mathrm{ATR_p}]$	Concentration of phosphorylated ATR	0.19
$[\mathrm{PHD_{tot}}]$	Total concentration of prolyl hydroxylase	0.56
$[\mathrm{PHD_a}]$	Concentration of activated prolyl hydroxylase	0.32
$[\mathrm{HIF\text{-}1}\alpha]$	Concentration of inactive HIF-1α	0.1
$[\mathrm{HIF\text{-}1}\alpha_{\mathrm{ac}}]$	Concentration of active HIF-1α	0.11
[p53]	Concentration of inactive p53	0.30
$[\mathrm{p53_p}]$	Concentration of phosphorylated p53	0.17
$[\mathrm{p53_{pac}}]$	Concentration of active p53	0.17
$[\mathrm{Mdm2_c}]$	Concentration of cytoplasmic Mdm2	0.0
$[\mathrm{Mdm2_{cp}}]$	Concentration of phosphorylated cytoplasmic Mdm2	0.125
$[\mathrm{Mdm2_n}]$	Concentration of nuclear Mdm2	0.06
$[\mathrm{Mdm2_{np}}]$	Concentration of phosphorylated nuclear Mdm2	0.023
[PUMA]	Concentration of PUMA	0.07
[p21]	Concentration of p21	0.08
[miR-17-92]	Concentration of miR-17-92	1.3
[PTEN]	Concentration of PTEN	0.22
$[\mathrm{Akt_p}]$	Concentration of active Akt	1.35
[PIP3]	Concentration of PIP3	0.6
[PIP2]	Concentration of PIP2	0.4
[FOXO]	Concentration of FOXO	0.08
[BIM]	Concentration of BIM	0.3
[CytoC]	Concentration of cytochrome c	0.06
[Casp3]	Concentration of active Caspase-3	0.05

SUPPLEMENTAL TABLE S2: PARAMETERS OF THE MODEL

Parameter	Description	Value	References
[p300]	Concentration of p300	1.0	Estimated
k_{shif}	Basal induction rate of HIF-1α	0.013	Estimated
$k_{\mathrm{dhifo_2}}$	$\mathrm{PHD_a}$-dependent degradation rate of HIF-1α	0.2	Estimated
$j_{\mathrm{hifo_2}}$	Michaelis constant of HIF-1α	0.5	Estimated
j_{o2}	Threshold of O_2 for PHD activation	5	Estimated
k_{sphd0}	Basal induction rate of $\mathrm{PHD_{tot}}$	0.001	Estimated
k_{sphd}	HIF-1α_{ac} inducible production rate of $\mathrm{PHD_{tot}}$	0.006	Estimated
j_{sphd}	Michaelis constant of HIF-1α_{ac} inducible $\mathrm{PHD_{tot}}$ production	0.3	Estimated
k_{dphd}	Degradation rate of $\mathrm{PHD_{tot}}$	0.002	Estimated
k_{acphd}	Activation rate of PHD	0.2	Estimated
j_{acphd}	Michaelis constant of PHD activation	0.5	Estimated
k_{dephd}	Inactivation rate of $\mathrm{PHD_a}$	0.07	Estimated
j_{dephd}	Michaelis constant of $\mathrm{PHD_a}$ inactivation	0.1	Estimated
k_{achif}	Acetylation rate of HIF-1α	0.4	Estimated
j_{achif}	Michaelis constant of HIF-1α acetylation	0.5	Estimated
k_{dhif}	Degradation rate of HIF-1α	0.01	Estimated
k_{dachif}	Deacetylation rate of HIF-1α_{ac}	0.1	Estimated
j_{dachif}	Michaelis constant of HIF-1α_{ac} deacetylation	0.1	Estimated
k_{dmhif}	Mdm2-dependent degradation rate of HIF-1α	0.1	Estimated
j_{dmhif}	Michaelis constant of Mdm2-dependent HIF-1α degradation	0.1	Estimated
k_{dhifac}	Degradation rate of HIF-1α_{ac}	0.005	Estimated
k_{smdm20}	Basal induction rate of $\mathrm{Mdm2_c}$	0.005	Estimated
k_{smdm2}	p53-dependent transcription rate of $\mathrm{Mdm2_c}$	0.06	Estimated
j_{smdm2}	Michaelis constant of p53-dependent $\mathrm{Mdm2_c}$ production	1.0	[9,33]
k_{dmdm2}	Degradation rate of cytoplasmic Mdm2	0.02	[9,33]
k_{1mdm2s}	Dephosphorylation rate of cytoplasmic Mdm2	0.3	[9,33]
j_{1mdm2s}	Michaelis constant of Mdm2 dephosphorylation	0.1	[9,33]

k_{mdm2s}	Akt-dependent phosphorylation rate of cytoplasmic Mdm2	8.0	[9,33]
j_{mdm2s}	Michaelis constant of Akt-dependent Mdm2 dephosphorylation	0.3	[9,33]
k_{i}	Nuclear import rate of $\mathrm{Mdm2_{cp}}$	0.06	[9]
k_{o}	Nuclear export rate of $\mathrm{Mdm2_{n}}$	0.09	[9]
k_{dmdm2n}	Basal degradation rate of nuclear Mdm2	0.03	Estimated
k_{pmdm2}	ATR-dependent phosphorylation rate of $\mathrm{Mdm2_{n}}$	0.07	Estimated
j_{pmdm2}	Michaelis constant of ATR-dependent $\mathrm{Mdm2_{n}}$ phosphorylation	0.3	Estimated
k_{dpmdm2}	Dephosphorylation rate of $\mathrm{Mdm2_{np}}$	0.1	Estimated
j_{dpmdm2}	Michaelis constant of $\mathrm{Mdm2_{np}}$ dephosphorylation	1.5	Estimated
k_{acatr0}	Production rate of ATR	0.15	Estimated
k_{acatr}	Activation rate of ATR	1.0	Estimated
j_{acatr}	Michaelis constant of ATR activation	1.0	Estimated
$k_{\mathrm{o_2}}$	Threshold of O_2 for ATR activation	0.01	Estimated
k_{deatr}	Inactivation rate of ATR	0.4	Estimated
j_{deatr}	Michaelis constant of $\mathrm{ATR_{p}}$ inactivation	0.6	Estimated
$\mathrm{ATR_{tot}}$	Total concentration of all forms of ATR	2	Estimated
k_{sp53}	Production rate of p53	0.02	Estimated
k_{pp53}	ATR-dependent activation rate of p53	1.5	Estimated
j_{pp53}	Michaelis constant of ATR-dependent p53 activation	0.5	Estimated
k_{dpp53}	Dephosphorylation rate of p53	1.0	Estimated
j_{dpp53}	Michaelis constant of $\mathrm{p53_{pac}}$ dephosphorylation	1.5	Estimated
k_{dmp53}	Mdm2-dependent degradation rate of p53	0.1	Estimated
j_{dmp53}	Michaelis constant of Mdm2-dependent p53 degradation	0.1	Estimated
k_{dp53}	Basal degradation rate of p53	0.05	Estimated
k_{acp53}	Acetylation rate of $\mathrm{p53_{p}}$	0.4	Estimated
j_{acp53}	Michaelis constant of $\mathrm{p53_{p}}$ acetylation	0.7	Estimated
k_{dmp53p}	Mdm2-dependent degradation rate of $\mathrm{p53_{p}}$	0.02	Estimated
j_{dmp53p}	Michaelis constant of Mdm2-dependent $\mathrm{p53_{p}}$ degradation	1.0	Estimated
k_{dp53p}	Basal degradation rate of $\mathrm{p53_{p}}$	0.005	Estimated

k_{dacp53}	Deacetylation rate of $\mathrm{p53_{pac}}$	0.15	Estimated
j_{dacp53}	Michaelis constant of $\mathrm{p53_{pac}}$ deacetylation	0.2	Estimated
k_{dp53pac}	Basal degradation rate of $\mathrm{p53_{pac}}$	0.005	Estimated
k_{sp210}	Basal induction rate of p21	0.0008	Estimated
k_{sp21}	HIF-$1\mathrm{a_{ac}}$ inducible production rate of p21	0.018	Estimated
j_{sp21}	Michaelis constant of HIF-$1\alpha_{\mathrm{ac}}$ inducible p21 production	0.65	Estimated
k_{dp21}	Degradation rate of p21	0.01	Estimated
k_{spuma0}	Basal induction rate of PUMA	0.001	Estimated
k_{spuma}	$\mathrm{p53_{pac}}$ inducible production rate of PUMA	0.025	Estimated
j_{spuma}	Michaelis constant of $\mathrm{p53_{pac}}$ inducible PUMA production	1.3	Estimated
k_{dpuma}	Degradation rate of PUMA	0.015	Estimated
k_{s0mir17}	Basal induction rate of miR-17-92	0.002	Estimated
k_{smir17}	$\mathrm{p53_{pac}}$ and $\mathrm{p53_{p}}$ inducible repression rate of miR-17-92	0.025	Estimated
j_{smir}	Michaelis constant of $\mathrm{p53_{pac}}$ and $\mathrm{p53_{p}}$ inducible miR-17-92 repression	0.8	Estimated
k_{dmir17}	Degradation rate of miR-17-92	0.02	Estimated
k_{spten0}	Basal induction rate of PTEN	0.0025	Estimated
k_{spten}	miR-17-92 inducible repression rate of PTEN	0.028	Estimated
j_{spten}	Michaelis constant of miR-17-92-dependent PTEN repression	0.1	Estimated
k_{dpten}	Degradation rate of PTEN	0.02	Estimated
k_{acakt}	Phosphorylation rate of Akt	0.25	[9,33]
j_{acakt}	Michaelis constant of Akt phosphorylation	0.1	[9,33]
k_{deakt}	Dephosphorylation rate of $\mathrm{Akt_p}$	0.1	[9,33]
j_{deakt}	Michaelis constant of $\mathrm{Akt_p}$ dephosphorylation	0.2	[9,33]
$\mathrm{Akt_{tot}}$	Total concentration of Akt	1.5	[9,33]
k_{p2}	Phosphorylation rate of PIP2	0.1	[9,33]
j_{p2}	Michaelis constant of PIP2 phosphorylation	0.2	[9,33]
k_{p3}	PTEN-dependent dephosphorylation rate of PIP3	0.5	[9,33]
j_{p3}	Michaelis constant of PIP3 dephosphorylation	0.4	[9,33]
$\mathrm{PIP_{tot}}$	Total concentration of PIP2 and PIP3	1.0	[9,33]

$\mathrm{FOXO_{tot}}$	Total concentration of FOXO	1.0	Estimated
k_{afoxo}	Phosphorylation rate of FOXO	0.1	Estimated
j_{afoxo}	Michaelis constant of FOXO phosphorylation	0.5	Estimated
k_{dpfoxo}	Dephosphorylation rate of $\mathrm{FOXO_p}$	0.05	Estimated
j_{dpfoxo}	Michaelis constant of $\mathrm{FOXO_p}$ dephosphorylation	1.5	Estimated
k_{sbim0}	Basal induction rate of BIM	0.003	Estimated
k_{sbim}	FOXO inducible production rate of BIM	0.02	Estimated
j_{sbim}	Michaelis constant of FOXO inducible BIM production	0.8	Estimated
j_{smbim}	Michaelis constant of miR-17-92-dependent BIM repression	1.5	Estimated
k_{dbim}	Degradation rate of BIM	0.01	Estimated
k_{puma}	PUMA-dependent release rate of mitochondrial cytochrome c	0.5	Estimated
k_{bim}	BIM-dependent release rate of mitochondrial cytochrome c	1.2	Estimated
k_{accytoc0}	Basal release rate of mitochondrial cytochrome c	0.001	[9]
j_{cytoc}	Michaelis constant of cytochrome c dependent Caspase-3 activation	0.5	[9]
k_{decytoc}	Mitochondrial influx rate of cytochrome c	0.05	[9]
$\mathrm{CytoC_{tot}}$	Total concentration of cytochrome c	3.0	[9]
$\mathrm{Casp3_{tot}}$	Total concentration of Caspase-3	3.0	[9]
k_{accasp30}	Basal activation rate of Caspase-3	0.001	[9]
k_{accasp31}	Activation rate of Caspase-3	0.9	[9]
j_{casp3}	Michaelis constant of Caspase-3 dependent cytochrome c release	0.5	[9]
k_{decasp3}	Inactivation rate of Caspase-3	0.07	[9]

SUPPLEMENTAL TABLE S3: PARAMETER ROBUSTNESS ANALYSIS

Parameter	+10%		-10%		Parameter	+10%		-10%	
	L_{HIF}%	L_{p53}%	L_{HIF}%	L_{p53}%		L_{HIF}%	L_{p53}%	L_{HIF}%	L_{p53}%
k_{achif}	**21.0**	-0.1	**-17.9**	0.1	j_{achif}	-13.9	-1.2	**19.0**	1.2
k_{shif0}	**31.9**	0.4	**-23.6**	-0.6	k_{dhif}	-2.9	-0.1	3.1	0.1
k_{dachif}	**-16.3**	0.0	**23.1**	-0.1	k_{dhifp}	-1.5	0.0	1.6	0.0
k_{acp53}	-2.6	8.2	5.7	-9.5	j_{acp53}	-4.5	-5.4	4.9	5.9
j_{dmp53}	-2.3	-3.2	2.6	2.8	k_{dacp53}	4.5	-8.1	-2.6	8.4
k_{dp53}	0.9	-2.1	-0.8	2.2	k_{dp53p}	0.9	-2.1	-0.8	2.1
k_{dp53pac}	4.1	-7.6	-2.8	9.4	k_{pp53}	-5.1	4.9	7.4	-5.7
k_{sp53}	-3.8	**16.4**	10.5	**-15.0**	k_{dmp53}	1.4	-3.2	-1.2	3.4
k_{acatr0}	0.1	0.1	-0.2	-0.1	k_{acatr}	1.7	1.0	-2.0	-1.3
k_{deatr}	-2.0	-1.3	1.9	1.2	k_{smdm20}	-2.3	-0.5	2.4	0.5
k_{smdm2}	**-21.3**	-5.6	**33.5**	5.1	k_{dmdm2n}	5.7	1.1	-5.4	-1.2
k_{pmdm2}	**24.4**	4.0	**-18.9**	-4.8	k_{dpmdm2}	-11.2	-2.6	-8.1	-1.8
k_{s0mir17}	-6.1	-1.3	9.2	1.7	k_{smir17}	-1.0	-0.2	1.0	0.2
k_{dmir17}	9.5	1.8	-7.4	-1.6	k_{spten0}	3.2	0.6	-2.8	-0.6
k_{spten}	**23.0**	3.8	-11.8	-2.7	k_{dpten}	-12.5	-2.9	**35.0**	5.3

Notes: Each bold number indicates HIF-1α_{ac} and p53_{pac} is more sensitive to variation in this parameter.

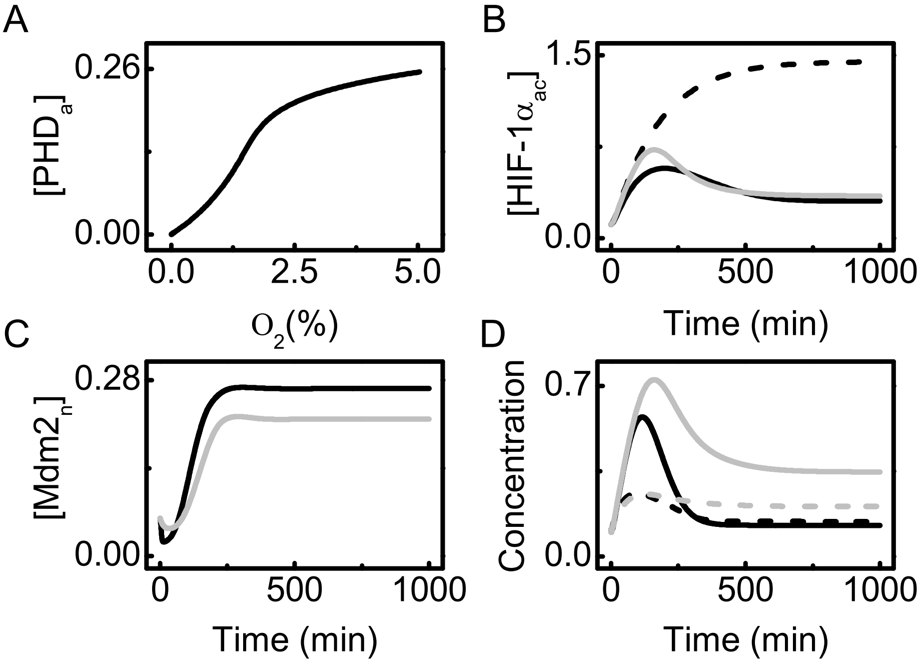

FIG. S1. (A) Bifurcation diagraphs of [PHD$_\text{a}$] as a function of O_2. (B) Time courses of [HIF-1α_ac] at $\text{O}_2 = 0.5\%$ (dashed), 0.02% (gray) or 0% (black). (C) Time courses of [Mdm2$_\text{n}$] at $\text{O}_2 = 0.02\%$ (gray) or 0% (black). (D) Time courses of [HIF-1α_ac] and [HIF-1α] at $\text{O}_2 = 0.02\%$ (gray and dashed, respectively) and 0% (black and dashed, respectively).

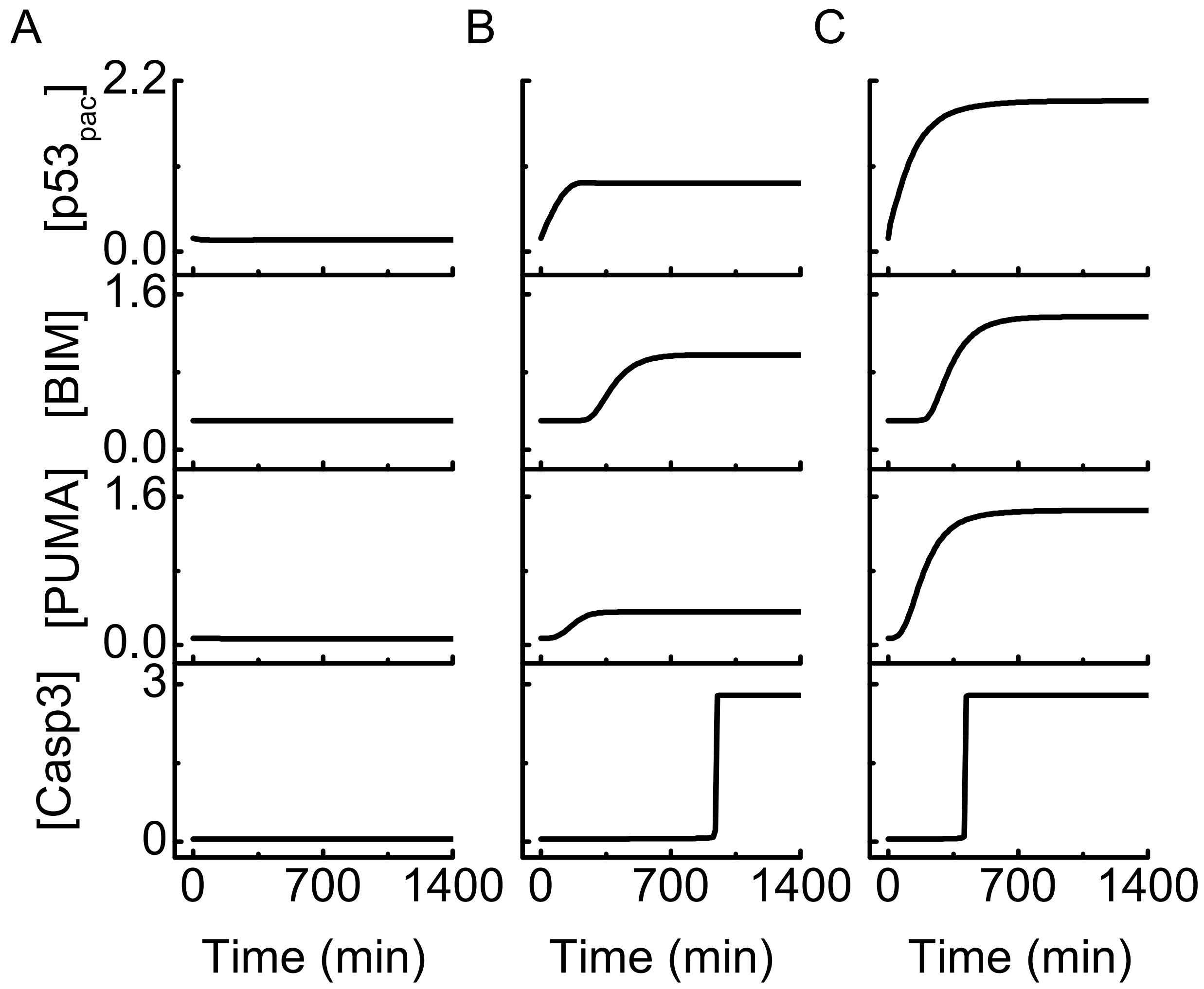

FIG. S2. Temporal evolution of the levels of $p53_{pac}$, BIM, PUMA and Casp3 under mild hypoxia (2%O_2, *A*), severe hypoxia (0.02%O_2, *B*) and anoxia (0%O_2, *C*).

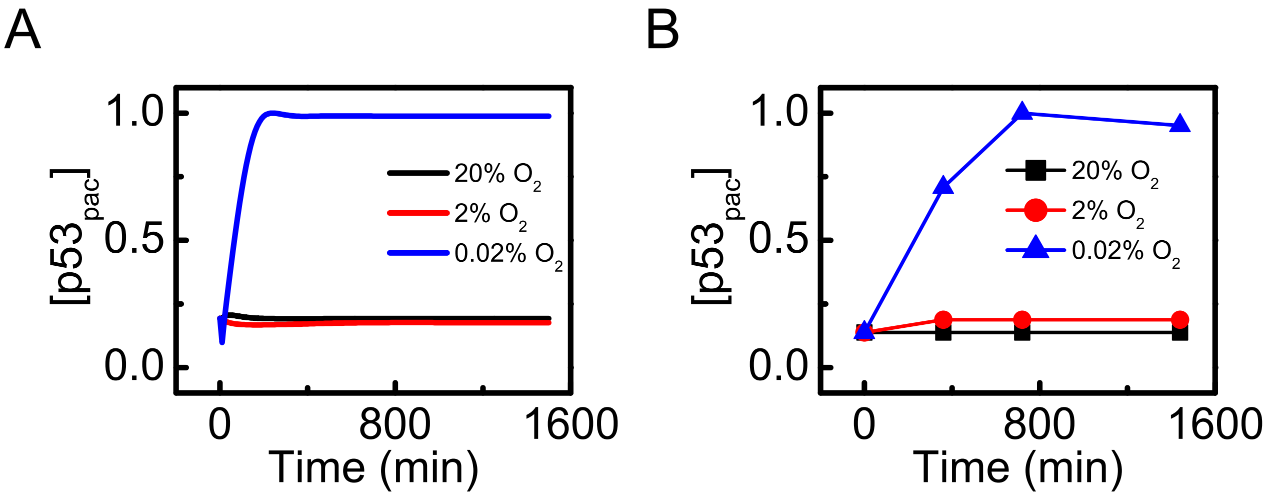

FIG. S3. Comparison of time courses of [p53$_{\rm pac}$] (A) with the experimental data (B) under various hypoxic conditions. The data are collected from Ref. 8 in the main text.
